# Visualizing the Topical Structure of the Medical Sciences: A Self-Organizing Map Approach

**DOI:** 10.1371/journal.pone.0058779

**Published:** 2013-03-12

**Authors:** André Skupin, Joseph R. Biberstine, Katy Börner

**Affiliations:** 1 Department of Geography, San Diego State University, San Diego, California, United States of America; 2 Cyberinfrastructure for Network Science Center, School of Library and Information Science, Indiana University, Bloomington, Indiana, United States of America; UMIT, Austria

## Abstract

**Background:**

We implement a high-resolution visualization of the medical knowledge domain using the self-organizing map (SOM) method, based on a corpus of over two million publications. While self-organizing maps have been used for document visualization for some time, (1) little is known about how to deal with truly large document collections in conjunction with a large number of SOM neurons, (2) post-training geometric and semiotic transformations of the SOM tend to be limited, and (3) no user studies have been conducted with domain experts to validate the utility and readability of the resulting visualizations. Our study makes key contributions to all of these issues.

**Methodology:**

Documents extracted from Medline and Scopus are analyzed on the basis of indexer-assigned MeSH terms. Initial dimensionality is reduced to include only the top 10% most frequent terms and the resulting document vectors are then used to train a large SOM consisting of over 75,000 neurons. The resulting two-dimensional model of the high-dimensional input space is then transformed into a large-format map by using geographic information system (GIS) techniques and cartographic design principles. This map is then annotated and evaluated by ten experts stemming from the biomedical and other domains.

**Conclusions:**

Study results demonstrate that it is possible to transform a very large document corpus into a map that is visually engaging and conceptually stimulating to subject experts from both inside and outside of the particular knowledge domain. The challenges of dealing with a truly large corpus come to the fore and require embracing parallelization and use of supercomputing resources to solve otherwise intractable computational tasks. Among the envisaged future efforts are the creation of a highly interactive interface and the elaboration of the notion of this map of medicine acting as a base map, onto which other knowledge artifacts could be overlaid.

## Introduction

Scholarly communication in the form of journal articles, book chapters, and related artifacts are the main means by which those engaged in the scientific process attempt to disseminate knowledge and ideas. Today, those engaged in extremely narrow scholarly pursuits may still be able to personally engage with most of the relevant literature by finding and reading individual articles, reflecting on them, drawing connections, and developing new ideas informed by their own education and experience. However, that is an increasingly rare scenario. Instead, processes of scientific knowledge construction and dissemination now occur within a multidisciplinary environment. In this new world of science, individual investigators *must* consider literatures from diverse disciplines. In addition, most cutting-edge science now occurs in multidisciplinary teams, whose members come from different academic backgrounds, with different intellectual cultures. The biomedical knowledge domain is a prime example, being a domain that is large, continuously growing, and with tremendously interdisciplinary make-up.

With the landscape of scientific inquiry thus increasingly opening up, and in consideration of the sheer number of scholarly articles in existence [1], the key question has emerged of *how* one might be able to analyze, present, and understand the complex, dynamic structures of science. Visualization-more specifically knowledge domain visualization-has been put forth as a powerful answer to that challenge [2], [3]. Apart from various examples of mapping individual domains and sub-domains, there has been increasing publicity around the general idea of science mapping, including through the traveling exhibit “Places & Spaces” (http://www.scimaps.org/) and the recently published “Atlas of Science” [2].

There has also been a renewed push to map “all” of science, with Klavans and Boyack [4] providing an overview of the various efforts. Among the more prominent examples are the “UCSD Map” based on five years of Web of Science and Scopus paper-level data available in the Science of Science Tool [5] and the international researcher networking tool VIVO (http://vivoweb.org/) or the paper-level, Web of Science based map that supports data overlays [6]. The medical knowledge domain has been prominently featured in such efforts as the NIH Visual Browser [7].

While the use of citation-based linkages dominates these science mapping efforts, online accessibility of publication databases has opened up other means for attempting to uncover the collective mind of scientists. An example is the use of click-streams that trace the sequence in which users navigate between online articles [8]. Whether maps are derived from such user interaction or from bibliometric structures, science mapping efforts are typically driven by a *network* conceptualization of science. Accordingly, network analysis and graph layout approaches dominate and a growing number of network-oriented toolsets can be utilized [9]–[11].

An alternative approach focuses on the *content* of documents, with an analysis of terms used by authors aimed at uncovering the topical structure and evolution of a domain. This tends to be based on a vector space representation of documents [12], where each document is represented by a vector of term weights. Those vectors can be used to compute document-to-document similarities and the resulting similarity matrix then be input to dimensionality reduction, with multidimensional scaling (MDS) a particularly popular approach [13]. However, the sheer size of similarity matrices limits the applicability of that approach for truly large data sets consisting of thousands or millions of documents. One popular alternative is the self-organizing map (SOM) method [14], which does not involve computation of similarity matrices. Instead, document vectors are interpreted as samples from a high-dimensional continuum [15] and are used to perform a tessellation of that original space such that it can be laid out in two dimensions. The result is a model that can itself be visualized or serve as a base map onto which high-dimensional vectors can be overlaid [16]. Numerous examples in applying this method to document vectors exist [17]–[20]. With respect to truly large data sets, Kohonen et al. [21] stands out, with a study involving several million patent abstracts.

The purpose of this paper is to elaborate on portions of a recent study whose aim was to investigate the accuracy of several text-based similarity approaches, for a data set of more than two million biomedical publications [22]. Specifically, we present a detailed explanation of how the SOM method was applied in the study, as well as several visualizations derived from the trained SOM. We also present a novel form of expert evaluation for such visualizations, with subjects providing both graphic and textual feedback to a large-format topical map of the medical sciences.

Our study differs significantly from previous work, in terms of the source and volume of data used, the techniques employed for creating document vectors, the specific SOM training method, the use of parallelization and supercomputing resources, the detailed elaboration of the principles and implications of the neuron label clustering technique, and the evaluation by subject experts. For example, previous work [20]involved a data set of 2,220 abstracts submitted to the Annual Meeting of the Association of American Geographers, with each abstract's title, author-chosen keywords, and full text used in the analysis, while the current project was focused on a data set larger by a factor of almost 1000, consisting of 2.15 million biomedical publications, with only MeSH terms being used in the analysis.

In previous work [19], [20], a vocabulary was generated from author-chosen keywords. That vocabulary was then used to generate a term-document matrix consisting of 2,220 documents and 741 term components, of which 1,148 documents are used for neural network training [19]. Meanwhile, the work described here generates a term-document matrix of 2.15 million documents and initially 23,347 MeSH terms, which is then reduced to the 2,300 most frequent terms whose occurrence is recorded for 2.14 million documents.

In the case of [19], [20], the input data set of 1,148 documents and 741 terms was used to train a self-organizing map consisting of 4,800 neurons. This was done using SOM_PAK [23]. The current project involved training of a SOM containing 75,625 neurons with a data set of 2.14 million documents represented as 2,300 dimensional vectors. In other words, the number of neuron weights in the current project is almost 50 times larger than in Skupin (2002, 2004) and the number of potential document weights is larger by a factor of almost 6,000. These dramatic differences required implementation of a batch variant of SOM, plus parallelization and deployment in a supercomputing hardware, which made it possible to train one of the largest self-organizing maps ever created in a non-hierarchical manner.

The work presented in [20] was centered on the use of different clustering techniques for helping organize large SOMs. Three clustering techniques were discussed and illustrated: hierarchical clustering, k-means clustering, and the neuron label clustering. While much of the current work is about exploring the latter, that technique played only a minor role in [20]. Specifically, the main figure showing a visualization of several thousand conference abstracts with simultaneous display of five cluster levels used hierarchical clustering ([20], p. 5275). Elsewhere, four different layers are combined, namely a term dominance landscape, k-means, hierarchical, and neuron label clustering (p. 5276). Incidentally, that is the only graphic depiction utilizing the latter technique, while the following figures contain either only hierarchical and k-means clustering (p. 5277) or only k-means clustering (p. 5278), respectively.

As the neuron labeling technique was only tangentially discussed in [20], the detailed discussion of term dominance profiles and of cluster patches resulting from neuron labeling clustering are other novel contributions made in the current work. The user evaluation by subject experts is likewise a new contribution for this kind of large-scale knowledge visualization.

## Methods

**Figure 1:**
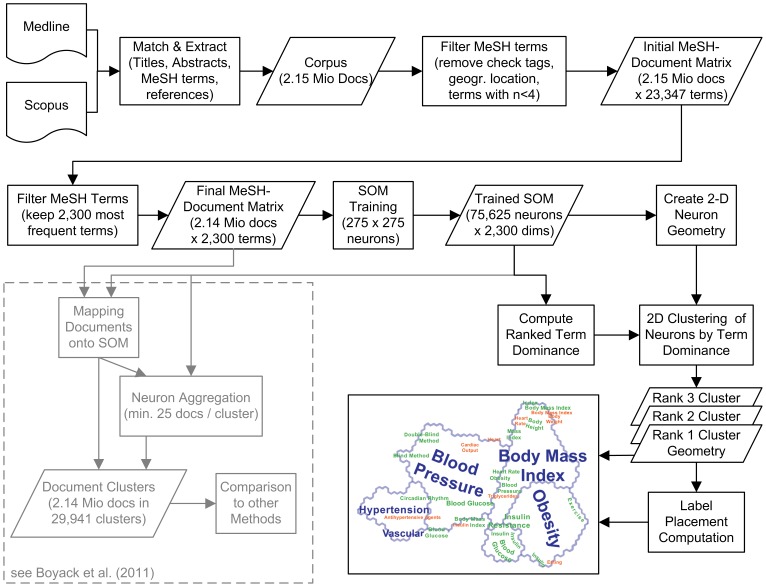
Processing steps for visualizing a large corpus of medical literature based on the self-organizing map method. The figure also references processing steps taken for the study by Boyack et al. (2011), which was centered on cluster quality.

Transforming a large document corpus into a compelling visualization requires a series of processing steps drawing on techniques that evolved in very different domains, ranging from computer science to information science, geography, and cartography (Figure 1). While the current study relies on identical source data and preprocessing steps as in [22], use of the self-organizing map method ultimately points toward a different underlying goal, namely the creation of low-dimensional geometric structures that allow leveraging the capabilities of the human perceptual and cognitive system for understanding a very high-dimensional space (see bottom-right of Figure 1). This contrasts with the goal of a strictly computational delineation of clusters within the document space (see bottom-left of Figure 1).

### Study corpus

As detailed in Boyack et al. [22], data from MEDLINE and Scopus were extracted to form a corpus of 2,153,769 documents published during a five-year period (2004–2008). All of those documents fulfilled that study's goal of containing abstracts, at least five MeSH terms (PubMed Medical Subject Headings), and at least five references.

### Text extraction and pre-processing

Among the document-specific elements included in the corpus, our study focused on MeSH terms. While references and the *network* structures that can be derived from them (e.g., co-citation links) are currently the dominant approach to the mapping of scientific communications, our approach is focused on a *topical* analysis of the biomedical knowledge domain. Titles, abstracts, and MeSH terms could provide the necessary data. Indeed, our study initially aimed at generating two topical models, one constructed from MeSH terms, the other from the text content of titles and abstracts, but with both models based on a representation of documents as high-dimensional vectors. In other words, using either data element (MeSH or title/abstract) the corpus would become represented as a vector space [12]. After filtering out MeSH terms and full text tokens appearing in under four documents (see [22] for details), the two initial data sets consist of 23,347 unique MeSH terms and 272,926 unique text tokens, respectively. For the MeSH case, the resulting vector space consists of binary entries indicating existence or absence of a term for each particular document. For the vector space constructed from title/abstract based tokens, actual counts of terms are recorded. Given the anticipated difficulty of dealing with data of such high dimensionality and the lower original dimensionality of MeSH-based vector space (n = 23,347), the study proceeded to focus on that data set first, with results reported in the remainder of this paper.

### SOM training

Since SOM training does not involve a computation of document-document similarities, the primary challenge in our study does not derive from the size of the document corpus as such, but rather from our goal of generating a detailed model of high-dimensional structures. This contrasts with the common use of self-organizing maps as a clustering tool, where neurons themselves act as clusters. For example, a SOM consisting of 10-by-10 neurons would generate a tessellation of the high-dimensional input space into 100 chunks, whose topological structure can then be visually explored. Meanwhile, our study does not primarily aim at clustering biomedical documents as such. Instead, the goal is a detailed two-dimensional layout of the high-dimensional space such that much of the inherent topical domain structure would be preserved. The principal means for accomplishing this is to generate a high-resolution SOM, i.e. a SOM consisting of a large number of neurons relative to the number of documents. For our corpus of 2.1 million documents, one would want to generate a SOM consisting of several hundred thousand neurons.

An initial attempt was made to generate such a model using the well-known toolkit SOM_PAK [23]. However, it soon became clear that modeling over 2 million documents on a suitably large map using SOM_PAK would not be computationally feasible, due to the concurrent conditions of large neuron count and large number of dimensions. Dimensionality of the input vector spaces is extremely high, whether dealing with title/abstract terms (272,926 dimensions) or MeSH terms (23,347 dimensions). Given its relatively lower dimensionality, the study focus shifted completely to the MeSH-based data set. However, use of its full set of terms was already deemed unfeasible. It was initially thought that reducing dimensionality by thresholding of MeSH term frequency would make deployment of SOM_PAK in a standalone PC-type environment feasible, as it had been in previous studies [20], [24], where models of up to 2,500 dimensions had been trained. However, even after generating a subset containing only the most frequent MeSH terms (2,300 dimensions) and limiting the number of neurons to fewer than 100,000, experiments on portions of the document collection suggested that complete training might require *years* of serial computation time.

**Figure 2:**
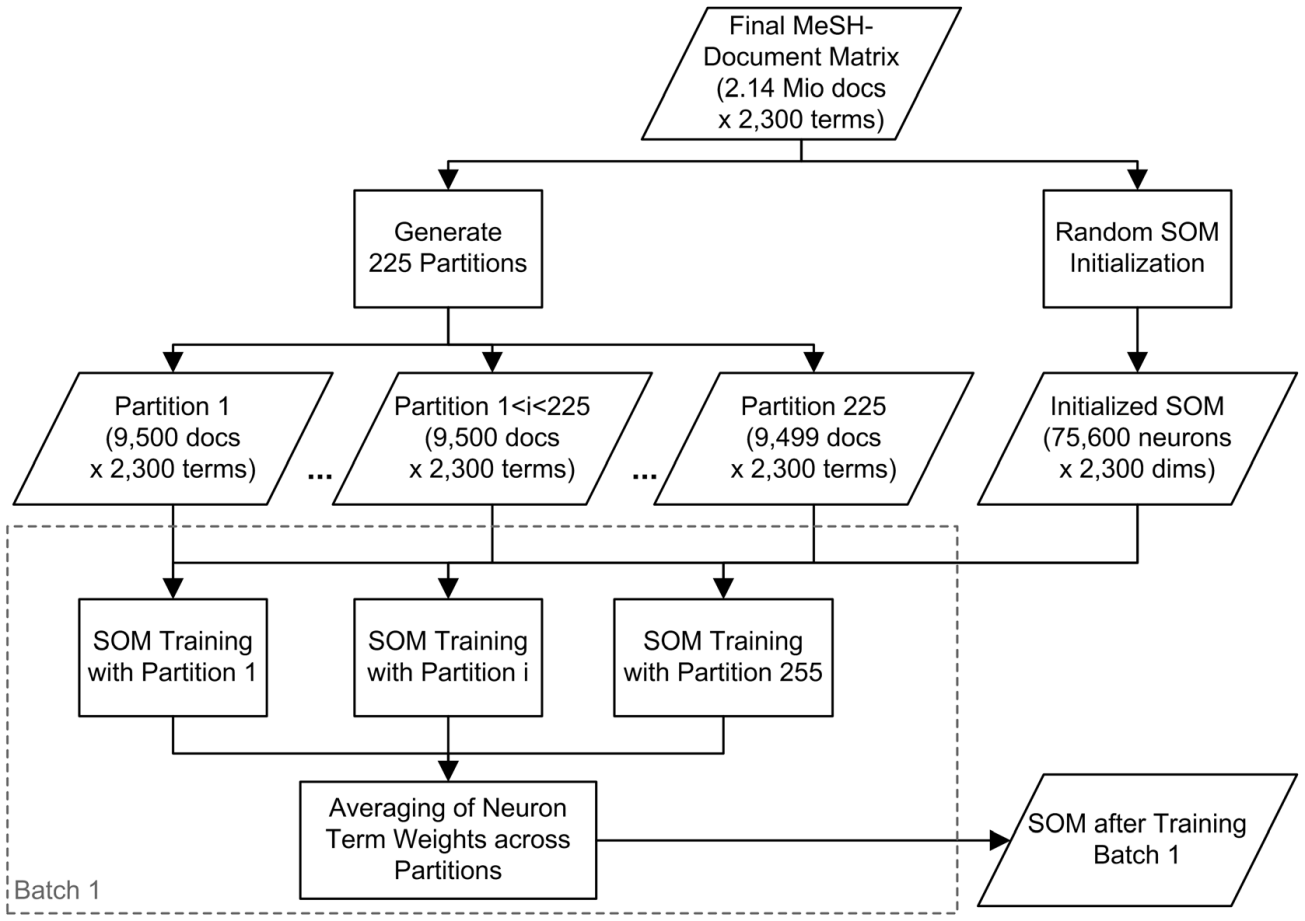
Parallelized batch training of the SOM, with 225 parallel processes. Included is only the first of a total of 240 sequential batches, with the trained SOM serving as input to the subsequent batch.

Keep in mind that although MeSH-based input vectors contain binary weights (i.e., presence or absence) and are very sparse (i.e., with few non-zero values for a given document), training will generate continuous weights for *all* dimensions at *each* neuron. Given the potentially small size of specific research communities, we also aim to use *all* documents during training, instead of relying on extensive sampling from the corpus. Consideration of those goals and of initial experiments made it clear that a parallelizable variation on the traditional SOM algorithm would be necessary to perform training with the entire document set in a reasonable amount of time. We implemented a particular “batch SOM” variant [25] and applied project- and infrastructure-specific optimizations to suit the data and available computational resources. In this “data-partitioned” batch SOM variant (Figure 2), the training data is divided equally among all processes and each process operates on a local set of neuron-specific accumulators representing pending updates to the map. At the end of each batch, all process-local accumulators are merged to apply that batch's global map update. This new map is then distributed back out to each process for the next batch of training.

With respect to further optimizing this process, we restructured the control flow to allow smaller batches, enabling more frequent process-global map synchronizations. Further, instead of re-calculating inter-neuronal grid distances at each accumulation, we traded time for space by calculating a single Gaussian kernel lookup table per batch. Finally, instead of the usual approach wherein each vector reflects the entire attribute space, we exploited the sparseness of the MeSH-document relation by characterizing each document as a collection of only the actually applied terms.

The following are core algorithmic elements of the training procedure:

- initialize each component of each neuron's reference vector randomly;
- initialize the new codebook per-neuron numerator and denominator accumulators to zero;
- initialize the width for the Gaussian neighborhood function to roughly equal the size of the map;
- until the start of the next batch, iteratively:
  - take the next training vector; search for the neuron with the nearest reference vector according to cosine similarity;
  - for each neuron, add to its accumulators a proportion of the training vector commensurate with the neuron's distance (determined by the Gaussian lookup table) from the neuron with the nearest reference vector.
  - If this is the end of a batch, combine the accumulators into a new live map, step down the width of the Gaussian, and reset the accumulators to zero.

The trainer consists of 1000 lines of C++ code. It relies only on standard language libraries and, for parallelization over an arbitrary number of processes, MPI.

### SOM post-processing

In addressing post-processing of the trained SOM, it helps to remember that the use of parallelization and supercomputing resources was necessitated by the goal of generating a fairly detailed low-dimensional model of a very high-dimensional space. The eventual use of a lattice of 275×275 neurons represents a compromise between the desire to capture detailed structures of the knowledge domain and the realities of contemporary computational resources. While we may have preferred to go much further in the spatial resolution of the SOM-yielding a lower document-to-neuron ratio-the actual SOM created does already go far beyond what is typical in SOM-based document mapping. Display of individual neurons (and of the individual documents captured by them) will be the exception and-in an interactive visualization-occur only after significant zooming to the local scale, while global and regional scale displays require significant computational and semiotic transformations of the original SOM into aggregate views. The foremost aim of our study is to generate a single, large-format visualization that simultaneously encompasses global and regional structures of the medical knowledge domain. Accordingly, the central post-processing step in dealing with a SOM of this size is the generation of neuron clusters, which includes partitioning the neuron space and determining appropriate label terms for such clusters.

This study utilizes a *neuron label clustering* method [20]. Though the binary weights associated with the 2.1 million input document vectors merely account for the occurrence or absence of terms, SOM training results in continuously scaled term weights. For each neuron, our clustering approach first computes the proportion of each term weight relative to the sum of neuronal term weights. This is referred to as *term dominance*. Terms within each neuron are then ranked in order of term dominance (see Figure 1). The highest weighted term becomes the top label term for that neuron. Next, if two neurons are neighbors in the two-dimensional neuron lattice and they share the same top-ranked label term, then their boundary is dissolved, thus forming a larger polygon, a neuron label cluster. Since SOM training tends to preserve topological relationships existing in high-dimensional space, neighboring neurons will have a good chance of being dominated by the same terms and thus the process of neuron aggregation can lead to fairly large 2D clusters.

As with any clustering approach, this tessellation of the input space is followed by the labeling of clusters. This tends to be a difficult task in knowledge visualization, since it requires extracting essential characteristics of cluster content, keeping in mind that these have to be communicable in a compact, meaningful form. For example, in a typical scenario, one would have to come up with one or two terms that succinctly summarize a cluster of dozens or hundreds of documents. Luckily, determining the cluster label is trivial with the clustering technique employed in this study, since a *single* term drives the creation of a cluster. It therefore becomes that cluster's label term.

Clustering is independently repeated for several more term dominance ranks, thus resulting in separate cluster solutions for first-ranked terms, second-ranked terms, and so forth. These later become layers in the visualization.

While determining the *content* of a label for individual neuron label clusters is easy, computing its *placement* is an extremely challenging combinatorial task. This stems from competing goals of (1) clearly associating a label with the respective geometric object, (2) maintaining label legibility, and (3) imbuing labels with semiotic properties driven by the source data, e.g., making quantitative and qualitative differentiations among labeled objects. While these considerations apply to any visualization, the sheer number of possible labels to be simultaneously placed make our particular task exceptionally difficult. In this study, the Maplex extension to the geographic information system (GIS) software ArcGIS is used. Maplex provides numerous controls, aimed at avoiding placement conflicts. We create label solutions in a hierarchical manner. First, label positions for the cluster solution computed from top-ranked terms are calculated, typically in fairly large font, in consideration of available display space. In order to avoid overprinting, those top-rank labels then constrain the labeling of second-rank neuron label clusters. Next, first- and second-rank cluster labels together constrain the placement of third-rank cluster labels, and so forth. In this manner, several ranked label solutions are built as layers that are all referenced to the same SOM and can thus be overlaid as desired.

Finally, cluster boundary geometry and labels are overlaid to form the actual visualization product. The geometry of only first-level clusters is depicted, plus its labels, and additional labels for other term dominance levels. Specifically, the map generated for examination by the subject experts contains geometry of first-level clusters, plus labels for three term dominance levels (Figure 1). Display of full cluster geometry for all cluster levels is precluded by the resulting clutter. However, we exploit the fact that intelligent label placement sometimes makes it possible to dispense with explicit display of feature geometry altogether, if labels manage to approximate that geometry in size and shape [26].

### Evaluation by subject experts

Interested in understanding the strengths and weaknesses of the SOM-based visualization, we conducted formal user evaluation using 10 domain experts. To our knowledge this is the first formal evaluation of a science map at this scale of number of publications and number of labels.

This study was accepted by the Human Subjects Office of Indiana University Bloomington as meeting the criteria of exempt research as described in the Federal regulations at 45 CFR 46.101(b), paragraph 2. The Human Subjects Office accepted the use of an information sheet. After reviewing the information sheet, all study participants gave verbal consent, which was recorded in a spreadsheet. Furthermore, completion of the study by each of the participants represented acceptance of consent.

The study comprises pre- and post-test questionnaires and hands-on map area identification tasks as detailed subsequently.

The pre-test questionnaire captured information on gender, age, native language, academic background, expertise, current employment, and familiarity with data/techniques of the study.

Next, each subject received an instruction sheet, a poster-size print of the map, and a red and a black marker. The instruction sheet provided brief information on the goals of the study, an introduction to the SOM method, and instructions on task at hand:

*“We are interested to increase our understanding of the strengths and weaknesses of a self-organizing map of more than 2 million MEDLINE publications we have made, and self-organizing maps in general.*

*Self-organizing maps create spatializations of high-dimensional input data, rendering many dimensions into a few that take advantage of the area available. The landscape of a self-organizing map is made up of many small cells, each of which has various levels of association with all of the terms in the term space. The self-organizing map is 'trained' so that the levels of association are based off how related terms are in the training documents, and neighboring cells have similar levels of association, generally. The landscape has peaks, where associations of the landscape with terms are strong, and valleys, where those associations are more tenuous. Terms can appear in multiple locations, especially if they have strong associations with multiple terms that are not themselves strongly associated.*

*To visualize a self-organizing map, areas of the map are labeled according to the terms that are most associated with an area. Lower ‘levels’ of terms, in this case represented by smaller text, are sub-terms in areas more strongly associated with terms represented in larger text. Peaks tend to occur where the terms you see are strongly associated together in meaning around an organizing topic. Valleys tend to occur where the terms have less strong associations, but are often related to the terms on nearby peaks. Associations on self-organizing maps are not just between neighbors. Self-organizing maps tend to have human-interpretable ‘regions’ of closely-related terms.*

*Please examine the large format self-organizing map of about 2,300 MeSH terms from two million MEDLINE papers. Please locate your own research area(s) on the map. Draw a boundary around them and label them in RED.*

*Identify six areas of science, draw a BLACK boundary around them, and label them. The areas can be as small as coaster or as large as a dinner plate. You don′t need to span the whole map, just try to identify main areas at similar levels of abstraction and give them a label that characterizes how them–it doesn′t have to be perfect.*

*This task will help us know how understandable the high-level structure of the map is.”*

## Results

### Quantitative characteristics of the model

A map of 75,625 neurons in a hexagonal lattice of 275 rows and 275 columns was randomly initialized, such that each of the 2,300 term MeSH term weights of each neuron took a value from {0, 1} with uniform probability. For each parallel process in each sequential batch, the width of the Gaussian kernel was dropped linearly from 250 down to 1 over the course of each batch. Training computations were carried out over three 2-day jobs across 225 processes running in parallel on the supercomputer Big Red at Indiana University. An equivalent serial runtime was estimated to require nearly 4 years. With a total of 108 million training time steps over 240 batches (each step involving a single input vector), the complete input data set of 2.1 million documents was presented to the training procedure over 50 times.

### Visualization

#### Visualizing the self-organizing map

In examining the various term dominance solutions (first rank, second rank, etc.), the sheer number of different terms rising to the top is surprising (Table 1). For example, 626 different terms appear as first-level label terms. In other words, more than 27 percent of 2,300 model terms are the highest-weighted term in at least one of 75,625 neurons. Further down the term dominance levels, a total of 94.3% of model terms rise to level 5 of term dominance. In other words, virtually all terms have a relatively high weight at least somewhere in the SOM. A number of factors contribute to this. One is of course the large number of neurons, which allows making finer distinctions within the knowledge space. This, in conjunction with NLM indexers choosing a very limited number of MeSH terms per document (mean: 7.06; median: 7.00; standard deviation: 3.67), produces a good chance that more terms have an opportunity to rise high in the rankings. That chance is even greater due to the elimination of all but the 2,300 most common MeSH terms early on.

**Table 1:** Statistics of label placement for top five term dominance levels.

Level	Unique Terms	Patch Count	Labels Placed	Labels Unplaced
1	626	818	606 (74.1%)	212 (25.9%)
2	1342	6126	1028 (16.8%)	5098 (83.2%)
3	1810	14936	1058 (7.1%)	13878 (92.9%)
4	2054	24649	899 (3.6%)	23750 (96.4%)
5	2170	32789	1056 (3.2%)	31733 (96.8%)

**Figure 3:**
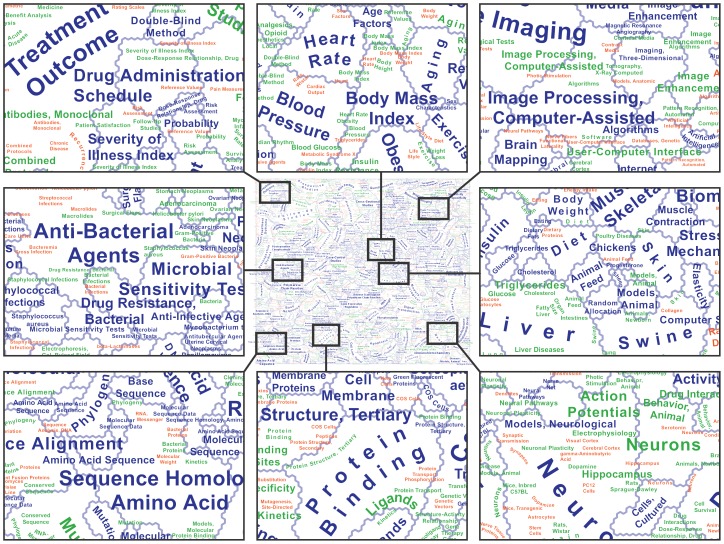
Zoomed-out view of the complete map of medical literature, plus detailed views of several regions. Contents and design as presented to domain experts for qualitative evaluation.

The first three levels correspond to the blue, red-orange, and green layers in Figure 3.

The explicit goal of the current study was to produce a map of the medical knowledge domain as large-format paper output. With a size of 30-by-36 inches for the core map-not counting legend and explanatory text-the result is difficult to convey in a journal paper. Figure 3 gives an idea of the look and content of the map, with a thumbnail version of the complete map in the center, surrounded by several detailed views. Notice the semiotic organization, with different term dominance rankings distinguished by color (blue: 1^st^ rank; green: 2^nd^ rank; red-orange: 3^rd^ rank) and cluster size indicated by label font size. Where appropriate and possible, labels attempt to follow the geometric shape of clusters. This is especially useful for second and third rank clusters, for which labels are the only possible indicator of geometry.

Variations in color, font size, and label geometry shape are meant to help with seeing meaningful patterns and relationships within a labeling solution that may initially seem enormously space-filling. What is not immediately apparent is that the number of labels appearing in the map is actually smaller than the number of contiguous neuron patches that *should* be labeled. At the top term dominance rank, only 606 out of 818 contiguous patches are labeled (74.1%), while the remaining 212 patches (25.9%) are not labeled (Table 1). Unplaced labels are caused by a confluence of small patch sizes, long label strings, and conflicts with the labels of neighboring patches. The number of unplaced labels rises dramatically for lower term dominance ranks, not only because the average patch size becomes lower. Another contributing factor is that higher-rank labels are used as exclusionary masks for lower-rank labels, in the interest of legibility. This additional constraint leads to 83.2% of second-rank patches remaining unlabeled, and by the time the system attempts to label fifth-rank patches, there is only enough space for labeling 3.2% of patches. Interestingly, the total number of labels placed remains fairly consistent from levels 2 onward, with between 899 and 1056 labels (Table 1), probably due to our effort of systematically reducing font sizes when labeling smaller neuron patches and lower term dominance ranks.

Contrary to many other clustering techniques, our approach does not constrain the total number of neuron patches. In our study, this results in a large number of patches (818 at the top level), but terms are by no means evenly distributed across those patches. One might expect that terms with a higher document frequency (Table 2) come to dominate a larger number of neurons (Table 3). However, that is not necessarily the case, since the list of terms with the highest neuron counts only partially matches the list of high-frequency terms. Notably, all matches between Tables 2 and 3 reflect fairly general, procedural concepts, such as these three siblings within the “Cohort Studies” category: “Follow-Up Studies”, “Prospective Studies”, “Retrospective Studies”. Meanwhile, high-frequency terms addressing broad groups of specific tools or techniques (e.g., “Molecular Sequence Data”, “Cells, Cultured”, “Amino Acid Sequence”) do not appear to dominate a correspondingly large number of neurons.

**Table 2:** The ten most frequent terms in the input data set, including the depth levels at which each term appears in the MeSH hierarchy.

MeSH Term	Doc Count	MeSH Level
Time Factors	137352	4
Treatment Outcome	132118	4/6/7
Molecular Sequence Data	100719	5
Risk Factors	95599	6/7/8
Retrospective Studies	94146	7/8
Follow-Up Studies	74821	8
Prospective Studies	72366	8
Cells, Cultured	71290	3
Sensitivity and Specificity	68620	3/5/6
Amino Acid Sequence	67764	5/6

**Table 3:** The ten terms occupying the most space in the map.

Top-Level Term	Neuron Count	Patch Count	Doc Count	MeSH Level
Follow-Up Studies	2328	2	74821	8
Treatment Outcome	2042	9	132118	4/6/7
Time Factors	1621	5	137352	4
Prospective Studies	1538	1	72366	8
Signal Transduction	1251	1	61715	4/5
Retrospective Studies	1170	5	94146	7/8
Questionnaires	1040	3	65557	4/5/6
Cell Line	1016	1	54657	4
Magnetic Resonance Imaging	1016	1	51776	5/6
Models, Biological	1015	1	58690	4

Terms are ordered according to the number of neurons for which each is the highest-weighted term. Also given is the number of contiguous patches for each term.

Looking at specific terms further clarifies that document frequency as such does not directly predict the area of the map dominated by a term (i.e., the neuron count). For example, while “Time Factors” occurs in almost twice as many documents as “Follow-Up Studies”, it is the top-ranked term in 31% *less* neurons. Meanwhile, “Molecular Sequence Data” is the third most frequent term in the input data, but does not make it into the top ten list at all, when neuron counts are considered.

It helps to consider that high-frequency terms may play different roles than simply dominating a *particular* research theme. Some high-frequency terms relate to *varied* research themes, such as when a popular technique is applied in different contexts. Using the term dominance technique, this might result in such terms being distributed across various map regions. As a result, such terms might form discontiguous regions or patches. Indeed, seven out of the ten most frequent terms make it onto the list of the ten terms with the highest patch count (Table 4). “Molecular Sequence Data” by far ranks at the top, with twice as many patches as the second-ranked “Risk Factors”. It seems that “Molecular Sequence Data” play a unique role in *very* varied circumstances, although in a secondary, supporting role that does not necessarily characterize what the local research theme is actually about.

**Table 4:** The ten terms occurring in the largest number of contiguous patches.

Top-Level Term	Patch Count	Neuron Count	Doc Count	MeSH Level
Molecular Sequence Data	20	699	100719	5
Risk Factors	10	1001	95599	6/7/8
Reproducibility of Results	10	517	64595	4/5/6
Treatment Outcome	9	2042	132118	4/6/7
Models, Molecular	8	275	48310	4
Amino Acid Sequence	6	904	67764	5/6
Time Factors	5	1621	137352	4
Retrospective Studies	5	1170	94146	7/8
Mutation	5	842	59174	4
Cells, Cultured	5	709	71290	3

Terms are ordered according to the number of patches. Also given is the number of neurons over which those patches are distributed.

#### Term Dominance Profile

**Figure 4:**
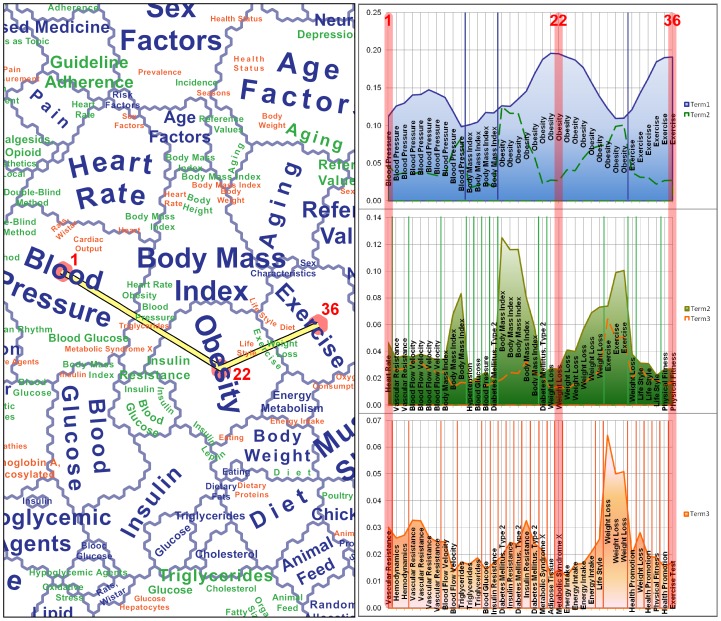
Transect through the term dominance landscape from “Blood Pressure” to “Exercise” via “Obesity”. Detailed profiles are shown for the first, second, and third-ranked terms for all transected neurons, with the line graph indicating the proportion of neuron vector weights accounted for by a particular label term. The first, last, and pivot neuron are highlighted (see also Table 5).

**Figure 5:**
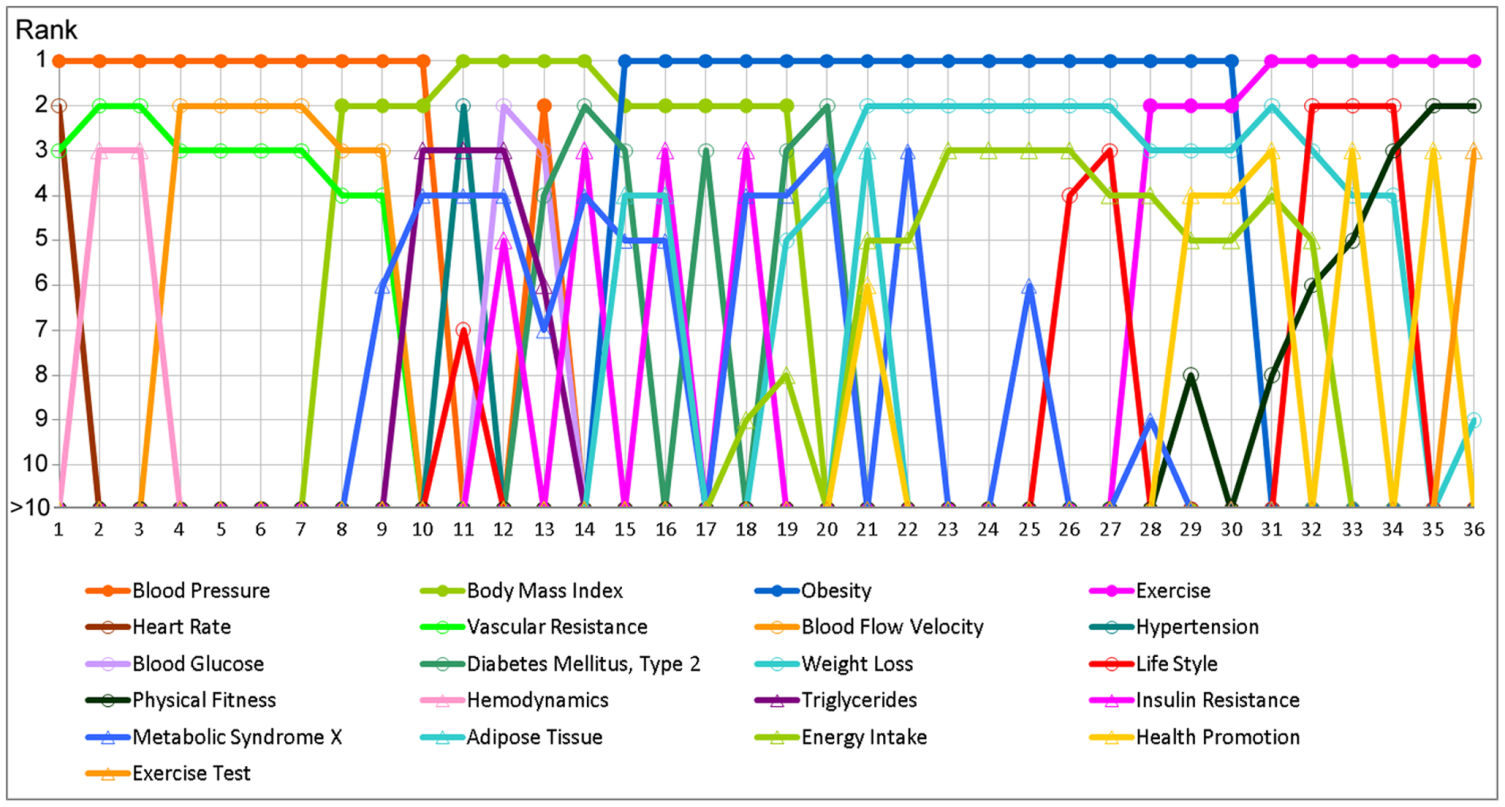
Term rank transitions along the 36 neurons transected in Figure 4. Included are all terms that make it to the first, second, or third term dominance rank in any neuron along the transect.

Though neuron label clustering was first discussed in [20], and has been used in a number of map projects, including Skupin's “In Terms of Geography” [2, p. 102–105], its specific construction and implications have never been explored in detail. Figures 4 and 5 and Table 5 are meant to fill that gap. We constructed a transect through the SOM, starting at a neuron near the center of the top-level region labeled “Blood Pressure”, crossing through a portion of the “Body Mass Index” region, making a turn within the “Obesity” region, and ending at a neuron within the “Exercise” region. In total, 36 neurons are traversed along the transect.

**Table 5:** Top ten terms for four neurons along the transect in Figure 4.

Rank	Neuron 1 (Blood Pressure)		Neuron 2 (Blood Pressure)		Neuron 22 (Obesity)		Neuron 36 (Exercise)	
1	Blood Pressure	0.1118	Blood Pressure	0.1259	Obesity	0.1952	Exercise	0.1908
2	Heart Rate	0.0470	Vascular Resistance	0.0351	Weight Loss	0.0268	Physical Fitness	0.0272
3	Vascular Resistance	0.0302	Hemodynamics	0.0260	Metabolic Syndrome X	0.0142	Exercise Test	0.0172
4	Regional Blood Flow	0.0183	Regional Blood Flow	0.0221	Dietary Fats	0.0113	Physical Endurance	0.0170
5	Vasodilator Agents	0.0141	Cardiac Output	0.0157	Energy Intake	0.0107	Physical Exertion	0.0105
6	Cardiac Output	0.0136	Sympathetic Nervous System	0.0107	Overweight	0.0081	Exercise Therapy	0.0081
7	Sympathetic Nervous System	0.0120	Ventricular Function, Left	0.0085	Body Composition	0.0057	Running	0.0054
8	Angiotensin II	0.0076	Vasoconstriction	0.0082	Health Policy	0.0057	Affect	0.0036
9	Vasoconstriction	0.0066	Vasoconstrictor Agents	0.0075	Food Habits	0.0054	Weight Loss	0.0033
10	NG-Nitroarginine Methyl Ester	0.0051	Pulmonary Artery	0.0063	Schools	0.0053	Task Performance and Analysis	0.0033

Table includes four neurons: the first neuron along the transect, its immediate neighbor, the pivot neuron, and the final neuron. For each neuron, terms are ranked according to their relative term dominance.

In Figure 4, three graphs express the term dominance of the first, second, and third-ranked terms along those 36 neurons. The four top-ranked regions are traversed in expected order (Figure 4, top right), with the first ten neurons having “Blood Pressure” as the term with the highest weight. For the next four neurons, “Body Mass Index” is dominant, and so forth. Term dominance- a term's weight divided by the sum of term weights for a particular neuron- for the top-ranked terms ranges from 10% to almost 20%, with fairly smooth transitions. Local maxima or peaks of term dominance are located near the centers of regions, while switches between top-ranked terms occurs near local minima.

The dominance values for second-ranked terms are prominently featured in the middle-right of Figure 4, but are also plotted as a dashed line in the top-right. It becomes clear that the centers of top-level regions are indeed clearly associated with particular terms (i.e., the second-ranked terms are trailing far behind), but that regional boundaries are less clear-cut, with first- and second-ranked terms having very similar term dominance.

In the graphs for the second- and third-ranked terms, notice how sharply the profile of term dominance values changes, as compared to the more smooth profile for the top-ranked terms. This has something to do with the nature of the neuron label clustering method and its ordinal ranking by term dominance and does not necessarily reflect absolute changes of term dominance for a particular term. For example, “Body Mass Index” gets demoted to second rank just inside the “Blood Pressure” and “Obesity” regions, though its absolute values remains quite high for a for a few more neurons.

In order to more fully explore dominance-based ranking of specific terms, Figure 5 contains rank transition profiles along the 36-neuron transect. For clarity, only terms that rise at least to third rank within the transect are included. Given the nature of the self-organizing map method and especially considering the level of detail supported by using tens of thousands of neurons, one would expect that terms slowly rise and drop in rank across the map. An example for the expected pattern is “Physical Fitness”, which slowly rises from rank 8 to rank 2 (see Figure 5) once the transect crosses the boundary between “Obesity” and “Exercise” (see Figure 4). A more surprising observation is that the weight and rank of some terms drop very dramatically and immediately near their local maxima. For example, “Obesity” immediately drops to below tenth rank once it is not first-ranked anymore. In other words, these are terms that are seen as worthy of succinctly characterizing a paper's overarching topic, but without value for characterizing either secondary meaning or specific research detail. Meanwhile, “Body Mass Index” is an example for a term that manages to remain in second-rank for a while on either side of its local transect maximum (behind “Blood Pressure” and “Obesity”, respectively), before dropping from sight (Figure 5).

Sometimes one observes rapid back-and-forth switching between certain terms, indicating that they are perhaps used synonymously by different researchers and as such are often used *near* each other (i.e., in otherwise closely related articles), but rarely within the *same* article. For example, notice how “Insulin Resistance” and “Diabetes Mellitus, Type 2” seem to play such a role between neurons 12 and 19. Though they clearly are related terms, it is surprising that they only once along this transect (in neuron 14) appear *together* among a neuron's top ten terms.

As one descends the term rankings within a neuron, one encounters finer semantic detail. The map itself attempts to express this through a visual hierarchy, especially via font size. However, labeling constraints make it difficult to perform a semantic drill-down for a particular location. Figures 4 and 5 help a bit in peeling back term layers for particular neurons. For example, for the 22^nd^ neuron along the transect (i.e., the pivot neuron), Figure 4 allows retrieving the rank sequence and relative dominance of the three terms “Obesity”, “Weight Loss”, and “Metabolic Syndrome X”. Figure 5 further reveals “Energy Intake” at rank 5.

One of the goals of developing SOMs with high resolution (i.e., large number of neurons relative to the volume of input data) is to enable exploration of semantic patterns at multiple scales. The *global* pattern along the shown transect has already been discussed, and *regional* patterns have been touched upon via second and third ranked terms (Figures 4 and 5). Beyond that, its is also possible to explore *local* patterns, i.e., the scale of specific research questions. Arguably, this is the scale at which most funded projects and project-specific research papers operate. Table 5 is meant to demonstrate how a deeper semantic drill-down may help in understanding those patterns. Included are the ten highest ranked terms for four neurons, namely (1) the first neuron along the transect and (2) its immediately neighboring neuron, (3) the pivot neuron, and (4) the last neuron of the transect. Neuron 1 and 2 are included in order to better appreciate the gradual transition of term weights. Notice how some terms appear to rise or fall in sync, such as observed with “Vascular Resistance”, “Cardiac Output”, and “Vasoconstriction”. Meanwhile, again, certain high-rank terms rapidly enter or leave the scene, such as when “Hemodynamics” appears to take the place of “Heart Rate”. Sometimes this process allows a term to rise in ranking despite a slight drop in dominance. Such is the case with “Sympathetic Nervous System”, whose rise along the transect seems to be enabled by “Heart Rate” and “Vasodilator Agents” simultaneously dropping out of sight.

**Figure 6:**
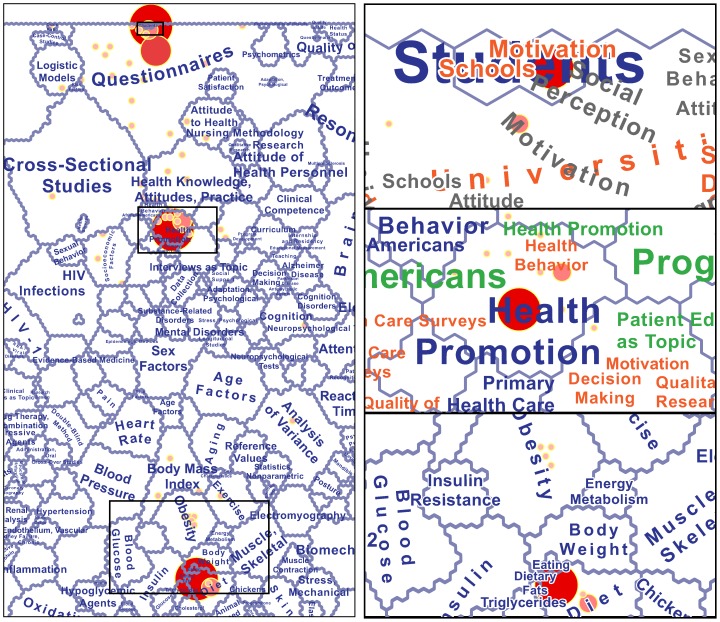
Base map function of the SOM demonstrated with an overlay of all articles containing MeSH terms "Food Habits" and "Schools". Larger circles indicate neurons with larger number of matching articles. Note the split into three main regions, each visualized at finer scale on the right.

As one approaches lower-ranked terms, one would expect that terms associated with very specific research questions come to the fore. That is because documents with matching low-ranked terms are likely to be closely related and perhaps indicative of a specific research theme. This should then drive local patterns in the SOM, i.e., patterns expressed through just a few neurons. Neuron 22 is a good candidate where a general focus on pathology (“Obesity”, “Metabolic Syndrome X”, “Overweight”) and physical factors (“Weight Loss”, “”Dietary Fats”, “Body Composition”) then turns towards broader, societal considerations (“Health Policy”, Food Habits”, “Schools”). This could be the starting point of further exploration of those research topics, like with a search for all articles containing the MeSH terms “Food Habits” and “Schools”, followed by a similarity-based overlay of those articles onto the base map of SOM neurons (Figure 6). In this example, it turns out that several relevant articles do in fact locate near the center of the “Obesity” region, but that the bulk of articles are found in three separate clusters, which could then be further investigated (see right portion of Figure 6).

Caution is advised when it comes to making more detailed local and document-level inferences that rely on lower-ranked terms, especially with ranks beyond the mean vector length of seven MeSH terms. The input data set contains plenty of documents with very few MeSH terms, including over 50,000 documents with only a single MeSH term and over 100,000 documents with two MeSH terms. One implication is that small variations in indexers' choice of MeSH terms can have dramatic effects on where a *single* document ends up getting placed. Note that this is different from the global and regional structures observed in the SOM, because these are the result of the training algorithm looking to preserve patterns contributed by *thousands* of documents. However, in order to support detailed exploration of local patterns, future extensions of this work should consider the full text of documents, yielding a richer, more nuanced data set.

### Results of expert subjects study

Formal user studies were run using ten subjects and the process outlined in section ‘Evaluation by subject experts.’ Here we report the results of pre-test questionnaire, hands-on map area identification and labeling exercise, post-test questionnaire, and debriefing session.

#### Pre-test questionnaire

Demographic attributes and expertise of participants varied as follows:

- Gender
  - Female: 3
  - Male: 7
-
  - Age:
    - 31–40 years: 3
    - 41–50 years: 2
    - 51–60 years: 2
    - 60+ years: 2
    - Unknown: 1
    - Native language
    1. English: 7
    2. French, Romanian, Spanish: one each
  -
    - Academic background:
  -
    1. Degrees ranging from B.S. to Ph.D. in areas as diverse as history, theology, sociology, biology, pathology, biophysics, social studies of science, information science (2), geology, clinical psychology, archaeology and law
  -
    - Expertise
      1. data analysis, medical research, cardiovascular medicine, genetics, biology (2), tissue engineering, bibliometrics, science policy (2), sociology of science, science education, geology, social science, environmental remediation, neuroscience, biochemistry, signal transduction, cell adhesion, psychopathology, psychophysiology, schizophrenia, electroencephalography, European prehistory, genomics and computational biology, domain mapping, law
    - Current employment:
      1. Program Analyst, Consultant, Senior Advisor, Senior Manager, Professor, and Librarian;
      2. Government (3), academic (6), combination of both (1);
    - Familiarity with data/techniques of the study
      1. MEDLINE (6)
      2. Medical Subject Headings-MeSH (4)
      3. self-organizing maps (2)

#### Hands-on map area identification and labeling exercise

**Figure 7:**
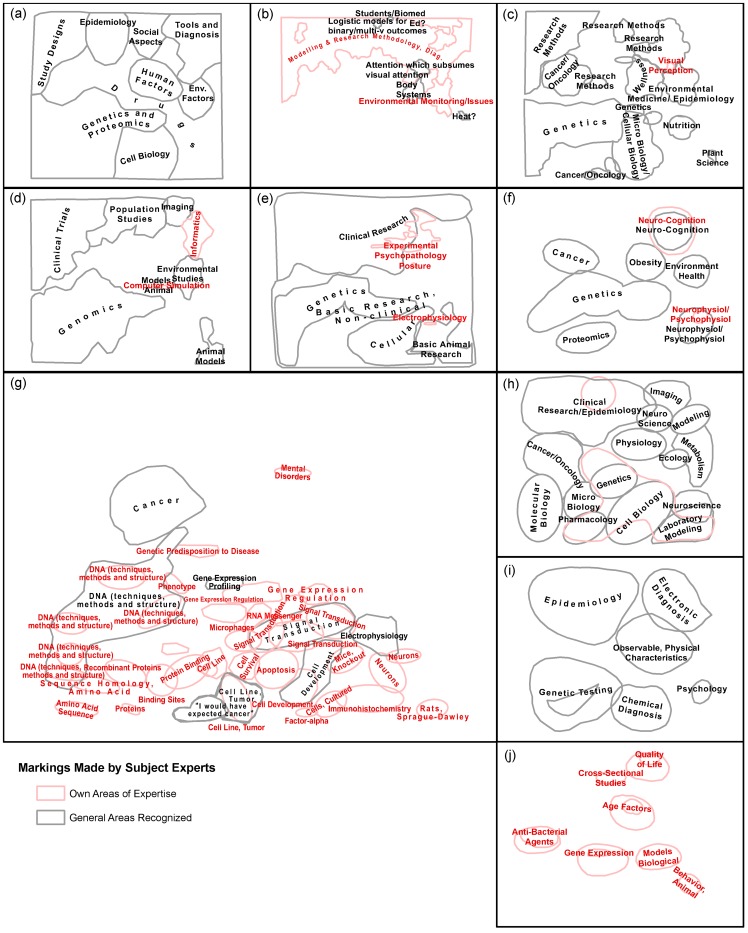
Digitization of markings made independently by ten subject experts on poster-size map printouts. Labels either explicitly stated by experts or extracted from encircled areas of the map. Subjects can be roughly categorized into Science of Science and information science researchers (a,b c), science analysts (d, e, f), and biomedical domain experts (g, h, i, j).

Here, subjects were asked to spent about 30 minutes to locate their own research area(s) on the map, draw a boundary around them, and label them in RED. Next, they were asked to identify six areas of science, draw a BLACK boundary around them, and label them. Each of the resulting ten annotated paper maps was digitized using a digital camera. Next, the subject-annotated markings for each map were vectorized on-screen and referenced to the corresponding digitized base map. The resulting maps are shown for comparison in Figure 7. As can be seen, some subjects perform no research in the biomedical domain that this map captures and their maps include no red areas. One subject (7(i)) has a degree outside of the medical sciences- which might explain why no red areas were delineated- but is now working in computational biology and genomics. Other subjects spent most of their time identifying the extensive areas of their own research and expertise, e.g., 7(b), 7(g), and 7(j). While identified areas and labeling differed considerably across maps, there are also a number of commonalities. For example, all but one subject identified and labeled the lower left to middle area as relevant for ‘Genetics,’ ‘Genomics’ or similar. In general, subject matter expertise seems to strongly impact the attention subjects pay to specific areas and labels on the map. For example, subject 7(g) focused mostly on areas close to his/her main area of expertise. Subjects 7(a) and 7(c) which had less biomedical expertise, produced rather similar areas and labels which is particularly surprising given the complexity of the map and the rather limited time frame to make sense of it.

Subjects verbally commented on the map during that part of the study: One domain expert pointed out that “‘Virology,’ Evolution,’ and ‘Paleontology’ are missing. ‘Virus replication,’ ‘HIV,’ ‘Adenovirus’ (gene therapy) should be close to ‘Virology.’” The subject also pointed out that “those terms do not line up with department names.”

Another pointed out that “there is a lack of terms that describe diseases. Many describe methodology which is typical for medicine. Could disease terms be extracted from documents? If researchers would get to pick terms: Would they pick diseases (treatment goals) or methodology terms (librarians seem method focused)?”

#### Post-test questionnaire

The feedback subjects provided in the post-test questionnaire is summarized in Table S1. In general, 30 minutes might be too little time to understand a map that aims to capture the semantic structure of more than two million documents. Most subjects were not familiar with MeSH and it might be hard to guess exactly which set of the 2300 MeSH terms used best characterizes a certain area. Some subjects expected to see methodology and technology labels while others expected disease term labels-this is most likely due to different expertise profiles but is also a reflection of the 2,300 most frequent MeSH terms covering mostly the top ranges of the MeSH hierarchy where specific methodologies/technologies/diseases are not yet encountered. Subjects were surprised/confused by label replications but most understood quickly that one method or object can be relevant for many models, diseases, etc. See also comments captured in debriefing session. Subjects were not sure if missing areas/labels were caused by limited data coverage (mostly biomedical research) or if they simply could not find a potentially lower level, small label without running an automated search over the map. There was also interest to interact with the data: to zoom in; to search for certain labels (e.g., “Clinical psychology”) on the map; and/or to display all documents matching a certain search query as symbols on the map, so that their placement can be understood. One subject suggested that we explain straight horizontal versus “warped” labels. S/he suggested using synthetic data and colors to explain layout, labeling, clustering, peaks and valleys on this abstract map. Two subjects suggested using contour lines or color coding to make peaks and valleys easier to infer.

#### Debriefing

Several subjects asked a number of additional questions and made diverse constructive suggestions during individual debriefing including:

- Were all 2300 terms active in all years?
- Would be nice to see all documents as point symbols.
- Different journals have different title lengths and number of keywords. They might have more general or more specific titles/keywords. How does this affect the map creation and reading?
- How many of the 2300 terms are used how often as labels and on what aggregation levels (different color and type fonts)? A frequency plot of label usage would be great.
- Please also provide distribution of MeSH terms over 2 million documents.
- What labels/MeSH terms are most spread out on the map-use TF-IDF? Which ones are frequent (across different levels) or clumped?
- Is there any correlation between the MeSH hierarchy and the clustering of MeSH terms on the map?
- Exactly how are area boundaries identified?
- Do (visible) lower level boundaries really hurt map reading?
- How to make valley's and hills more visible? Some had seen the LastFM map and seemed to prefer its rendering.
- Add numbers 1…275 on outer map boundaries to create a reference system?
- Spread out vs. normal writing/labeling; horizontal vs. warped writing; but also label density lead to different perceptual dominance of terms that are little understood but might have a major impact on map reading.
- What does type font represent? 3^rd^ level labels can be much larger than 1^st^ level-this is different from cartographic maps.
- New user study idea: Give subjects a small area on the map and have them generate more terms/labels for that area.

Some of these questions and comments are specifically addressed in this paper (see previous section). Others provide valuable input to further improve the usability/usefulness of the presented map and other science maps.

## Discussion

### Limited Vocabulary and Vector Sparseness

One of the goals of this project was to see how far the application of a basic vector space model in conjunction with standard SOM training could be pushed when faced with a truly large document corpus. That required a dramatic reduction of terms, using only the ∼10% most frequently occurring MeSH terms, which automatically reduces the semantic richness of any resulting model. Further, in relation to the total remaining vocabulary of 2,300 MeSH terms, the vectors representing individual documents are exceedingly sparse, with an average of only seven terms per document. That sparseness implies that there is less opportunity for multi-facetted overlapping of the content of vectors. As a result, the model is somewhat “flat”, lacking in semantic richness, as compared to previous SOM-based visualization examples [20]. This is especially true at the local level, where one might want to investigate relationships among individual papers. Meaningful local operations along the lines of “show me the ten most similar papers to the one I selected on the map” will require construction of more comprehensive document vectors, ideally from articles' full text. The most immediate alternative would be to use the already existing title/abstract terms and that is indeed one of the envisaged near-term extensions to this study.

### Limited Scale Range of Static Map

With a visualization derived from over two million documents, one should ideally be able to investigate patterns at a range of scales, from the global level (e.g., dominant groupings of topics) to the local level (i.e., relationships among individual documents). A static visualization, such as created in this project, does not lend itself to covering that full range. For the future, a highly interactive, zoomable interface, should provide meaningful scaling, based on appropriate computational approaches. Hierarchical clustering is one of the methods particularly suited to multi-scale visualization, due to the cognitively advantageous nesting of scale levels [19], [20]. Other methods would allow a statistically more optimal generation of scale-dependent patterns-like k-means clustering-but at the price of lacking coordination across scales [20]. Creation of a zoomable interface to the STS map would also depend on the type of rich, literally multi-layered, content that the current degree of term filtering does not provide.

### Topics as Dimensions in SOM

As stated before, much in this experiment was geared towards a methodologically straightforward and “clean” vector-space and SOM apparatus. With the help of supercomputing resources, the project was able to push this approach much further than would otherwise be possible. Though only 10% of MeSH terms were used, those were the most frequently used terms. Given the much lower frequency of the eliminated terms and the overall sparseness of term vectors, we would not have gained much semantic power by just moving up to using 20% or 30% of the MeSH terms, at the cost of a doubling or tripling of the model's dimensionality. Instead of reducing dimensionality by merely dropping dimensions, one should aim to combine all of the initial terms into aggregate/surrogate dimensions that then form the basis of a vector-space model of documents. Armed with such a reduced-dimensionality model, one can then proceed to train a SOM of even higher resolution than in the current experiment, with several hundred thousand or even a million neurons. Latent Dirichlet Allocation (LDA) would be a particularly interesting candidate, with its latent semantic topics serving as dimensions for further analysis. Compared to a typical LDA approach, one would aim to extract a relatively large number of topics (e.g., several hundred), which then become the SOM's dimensions. Use of a topic model also seems essential with respect to a move from MeSH keywords to handling full text data.

### Controlled Vocabularies

It appears that the use of indexer-chosen keywords, including in the case of a large controlled vocabulary-MeSH terms in this study-raises interesting questions. The rank transition diagram in particular helped to highlight the fact that different vocabulary items play different roles in indexers' attempts to characterize the content of specific publications. The complex interplay of hierarchical relationships and functional roles of MeSH terms deserves further investigation, which may inform future efforts of how specific terms are handled in computational analysis. For example, models constructed from terms occurring at intermediate levels of the MeSH hierarchy might look and function quite different from the top-level model presented here.

### User-centered Studies

Future user studies will include term differentiation tasks to help us understand whether/how users can differentiate senses of terms on the self-organizing map. When a term appears prominently in multiple places, that indicates multiple senses or contexts for that term. One study might involve subjects being shown two regions within which a particular label term appears and the abstracts of several papers containing that term. Subjects would then be asked to rate each abstract along a continuum between two extremes formed by the two senses/contexts. Studies like that will help us evaluate how understandable the local structure of the map is.

### Exploratory Visual Analysis

This paper includes numerous examples of how a detailed SOM-based representation of a very large document corpus could be further explored, including use of transects, profiles, rank transition diagrams, and drill-downs. All of these build on the nature of the SOM method, with its conceptualization of a continuous high-dimensional space that is then represented in tessellated, low-dimensional form. This draws a distinction to previous efforts of representing document spaces based on layered *density* landscapes [11], [27], [28] that ultimately are driven by a discrete object conceptualization [24].

### Interactive Map

**Figure 8:**
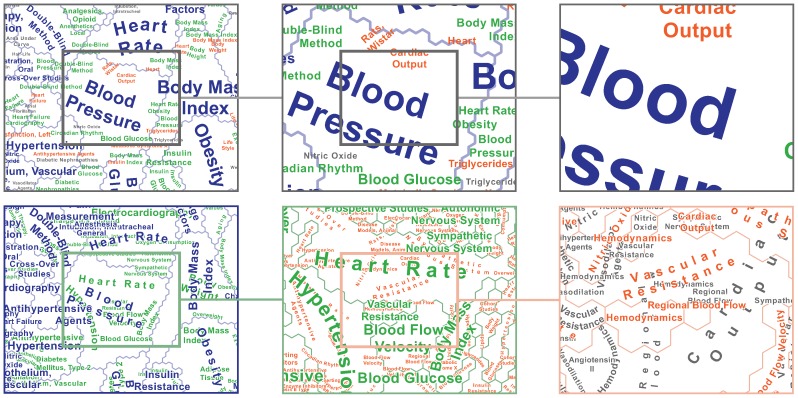
Geometric zooming versus semantic zooming. Juxtaposed are examples of geometric zooming into the static display of multiple levels optimized for preventing label overlaps (top) versus semantic zooming with successive revealing of lower levels of term dominance (bottom).

An obvious direction for future work is to transform the static, print-focused map of the biomedical knowledge domain into a highly interactive Web-accessible application that would support such functions as search, selection, and scale change (i.e., zooming), as requested by several human subjects. Some of the enormous constraints imposed by the conjunction of a single static display with sheer corpus size could be loosened in an interactive setting. For example, it would become possible to aim for semantic zooming, with detailed concepts being revealed as users zoom in. Added interactivity would allow more freedom in simultaneous display of multiple levels, revealing more of the semantic richness of the document space (see sequence of zooms in Figure 8), as compared to the large numbers of patches remaining unlabeled in the current map (Table 1).

Extending the target user group of such an interactive application beyond the core biomedical community will require significant enhancements. For example, users searching for “Cancer” in the current map might be surprised that the term is virtually absent. Meanwhile, “Neoplasms” and various related noun phrases (e.g., “Breast Neoplasms”, “Antineoplastic Agents”) are MeSH terms associated with tens of thousands of documents in the corpus and large swaths of the map. Making the map truly accessible to the general public or out-of-domain researchers will require exploiting ontological links between expert-preferred terms and laypersons' language.

## Supporting Information

Table S1 Subject responses.
(XLS)
